# Circulating levels of IL-1 family cytokines and receptors in Alzheimer’s disease: new markers of disease progression?

**DOI:** 10.1186/s12974-018-1376-1

**Published:** 2018-12-12

**Authors:** Paola Italiani, Ilaria Puxeddu, Sabrina Napoletano, Emanuele Scala, Daniela Melillo, Simone Manocchio, Antonella Angiolillo, Paola Migliorini, Diana Boraschi, Emilia Vitale, Alfonso Di Costanzo

**Affiliations:** 1 Laboratory of Innate Immunity and Inflammation, Institute of Protein Biochemistry, National Research Council, Via Pietro Castellino 111, 80131 Naples, Italy; 2 Clinical Immunology Unit, Department Clinical and Experimental Medicine, University of Pisa, Via Roma 67, 56126 Pisa, Italy; 3 NeurOmics Laboratory, Institute of Protein Biochemistry, National Research Council, Via Pietro Castellino 111, 80131 Naples, Italy; 4 Centre for Research and Training in Medicine for Aging, Department of Medicine and Health Sciences “Vincenzo Tiberio”, University of Molise, Località Tappino, 86100 Campobasso, Italy

**Keywords:** Alzheimer’s disease, Mild cognitive impairment, Subjective memory complaints, IL-1 family, Cytokines, Receptors, Inflammation

## Abstract

**Background:**

Although the mechanisms underlying AD neurodegeneration are not fully understood, it is now recognised that inflammation could play a crucial role in the initiation and progression of AD neurodegeneration. A neuro-inflammatory network, based on the anomalous activation of microglial cells, includes the production of a number of inflammatory cytokines both locally and systemically. These may serve as diagnostic markers or therapeutic targets for AD neurodegeneration.

**Methods:**

We have measured the levels of the inflammation-related cytokines and receptors of the IL-1 family in serum of subjects with AD, compared to mild cognitive impairment (MCI), subjective memory complaints (SMC), and normal healthy subjects (NHS). Using a custom-made multiplex ELISA array, we examined ten factors of the IL-1 family, the inflammation-related cytokines IL-1α, IL-1β, IL-18, and IL-33, the natural inhibitors IL-1Ra and IL-18BP, and the soluble receptors sIL-1R1, sIL-1R2, sIL-1R3, and sIL-1R4.

**Results:**

The inflammatory cytokines IL-1α and IL-1β, their antagonist IL-1Ra, and their soluble receptor sIL-1R1 were increased in AD. The decoy IL-1 receptor sIL-1R2 was only increased in MCI. IL-33 and its soluble receptor sIL-1R4 were also significantly higher in AD. The soluble form of the accessory receptor for both IL-1 and IL-33 receptor complexes, sIL-1R3, was increased in SMC and even more in AD. Total IL-18 levels were unchanged, whereas the inhibitor IL-18BP was significantly reduced in MCI and SMC, and highly increased in AD. The levels of free IL-18 were significantly higher in MCI.

**Conclusions:**

AD is characterised by a significant alteration in the circulating levels of the cytokines and receptors of the IL-1 family. The elevation of sIL-1R4 in AD is in agreement with findings in other diseases and can be considered a marker of ongoing inflammation. Increased levels of IL-1Ra, sIL-1R1, sIL-1R4, and IL-18BP distinguished AD from MCI and SMC, and from other inflammatory diseases. Importantly, sIL-1R1, sIL-1R3, sIL-1R4, and IL-18BP negatively correlated with cognitive impairment. A significant elevation of circulating sIL-1R2 and free IL-18, not present in SMC, is characteristic of MCI and disappears in AD, making them additional interesting markers for evaluating progression from MCI to AD.

**Electronic supplementary material:**

The online version of this article (10.1186/s12974-018-1376-1) contains supplementary material, which is available to authorized users.

## Background

Alzheimer’s disease (AD) is an age-related, non-reversible brain disorder that develops over a period of years and has become a global challenge for public health. Currently, over 40 million people worldwide live with AD, and this number is expected to double by 2030 [1].

AD is characterised by the loss of neurons and synapses in different areas of the brain. Usually AD manifests first with a mild cognitive impairment (MCI) before progressing to frank dementia in which memory impairment is the most prominent cognitive deficit. Histological hallmarks of AD are an extracellular deposition of plaques generated by sequential proteolytic cuts of the amyloid precursor protein, and an intracellular accumulation of neurofibrillary tangles produced by abnormal aggregation of hyperphosphorylated protein tau. The most prominent risk factor for AD is age, and 65 years represent the benchmark for discrimination between early onset (about 10% of cases, with a likely genetic cause) and late onset (90% of cases, with multiple risk factors) [2]. Despite intensive research and drug development efforts, there are still no therapies that can stop or even slow down disease progression.

Extensive data show that a low-grade chronic inflammatory response is present in AD, suggesting that inflammation may be involved in AD-associated neurodegeneration [3]. Increased levels of inflammatory mediators, in particular C-reactive protein and IL-6, have been found in the brain and in cerebrospinal fluid (CSF) of AD patients and correlate with an increased risk of dementia [4], thus suggesting a microglial-based inflammatory state in the AD brain [5]. Postmortem analysis of AD brains has provided evidence for pathology-associated inflammation. In fact, the inflammatory cytokines IL-1β, IL-6, and TGF-β, among others, were found accumulating around the amyloid plaques in the brain of AD patients [6–8]. In line with these results, numerous studies focused on analysis of the presence and levels of inflammatory and anti-inflammatory cytokines in the CSF and serum of patients with MCI or AD. It seems that both inflammatory (IL-1β, IL-6, TNF-α) and anti-inflammatory cytokines (the IL-1 receptor antagonist IL-1Ra, IL-10) are elevated in CSF and circulation of AD patients [5]. In the AD brain, it is hypothesised that microglial cells are activated by amyloid plaques and neurofibrillary tangles, detected as potential dangers, and initiate an inflammatory reaction with the production of inflammatory mediators such as cytokines and chemokines [9, 10], a reaction that becomes chronic due to the persistent presence of plaques and tangles.

MCI is characterised by a cognitive decline that exceeds normal age-associated changes, and represents an intermediate stage between normal cognitive functions and dementia. The rate of progression of MCI to dementia is estimated to be 10–15% annually, with up to 46% of MCI patients developing dementia over 3 years [11]. Thus, MCI may be considered the preclinical stage of dementia and represents a reliable predictive marker [12]. Among the many cognitive changes observed, impairment in episodic memory is most commonly seen in MCI patients who subsequently progress to a diagnosis of AD, which is the most common type of dementia [13]. Subjective memory complaints (SMC) indicate a self-reported memory decline, which may or may not be perceived by others, in the absence of pathological results in neuropsychological tests. Subjects diagnosed with SMC have an increased risk of progressing to MCI (26.6%) and dementia (14.1%) within 4 years [14].

Several studies have examined the activation of inflammatory pathways as a pathological hallmark in MCI and AD, with the goal of identifying both markers of progression from MCI to AD and possible therapeutic targets, although the results obtained are controversial or inconsistent [5, 15–17].

Among inflammation-related factors, the cytokines and receptors of the IL-1 family are particularly important for their ability to activate and regulate inflammatory responses [18, 19].

The highly inflammatory cytokine IL-1β, and its sister cytokine IL-1α, are usually present in circulation only at very low levels [20], most likely because of their dangerous vasoactive effects. The activity of IL-1β and IL-1α is effectively regulated by a number of decoy receptors and soluble antagonists. IL-1α and β bind to the receptor IL-1R1, which forms a signalling complex with the accessory chain IL-1R3. IL-1R1 can also bind the antagonist IL-1Ra, which blocks the formation of the signalling complex thereby inhibiting IL-1-induced receptor-mediated inflammatory activation. The extracellular IL-1R1 domain, when not membrane-bound (soluble IL-1R1, sIL-1R1), can capture IL-1 cytokines in solution, acting as a soluble decoy. The second receptor for IL-1, IL-1R2, binds IL-1β with high affinity and recruits IL-1R3, but does not initiate intracellular signalling, also acting as a decoy receptor. It has been shown that the soluble form of IL-1R3 increases the affinity of soluble IL-1R2 in capturing/inhibiting IL-1β. The tight regulation of IL-1 activity underlines its potent inflammatory effects, which must be closely controlled to avoid pathological derangements. Excessive IL-1 activity underlies a wide range of inflammation-related diseases, a notion supported by the effectiveness of anti-IL-1 therapy with Anakinra (recombinant IL-1Ra) or Canakinumab (anti-IL-1β antibody) [21, 22]. The role of both IL-1α and IL-1β in brain damage and neuroinflammation has been vastly investigated, with IL-1 involved in inflammation-related neuronal damage and neurodegeneration, but also taking part in the mechanisms of learning and memory (for a review, see [23]). In AD, microglial IL-1 production and some polymorphisms in the *IL1* genes have been found to correlate with disease both in humans and in experimental models [23], although after a meta-analysis of data no association between *IL1* loci and AD was evident [24].

Another important cytokine of the IL-1 family is IL-18, a factor with both inflammatory effects and metabolic/immunological regulatory activities [25, 26]. IL-18 activity is downregulated by the IL-18-binding protein (IL-18BP), a soluble receptor-like protein that binds IL-18 with high affinity and inhibits its interaction with cell surface receptors and consequent cell activation. IL-18 induces the production of IFN-γ, which is the most potent stimulus for the production of IL-18BP, thus self-regulating its own activity through the indirect induction of its inhibitor [26, 27]. Measurable concentrations of IL-18 are detectable in sera of normal subjects, yet are increased in many pathological conditions [26, 27]. In pathological conditions in which both IL-18 and IL-18BP were measured, such as amyotrophic lateral sclerosis (ALS), systemic lupus erythematosus (SLE), and IgG4-related disease, the higher levels of circulating IL-18 are paralleled by increased circulating IL-18BP, most likely as an attempt to counteract the excess of IL-18 [28–33]. Similar to IL-1, IL-18 in AD appears to be a sign of increased inflammation, with higher levels of circulating IL-18 in AD patients, accumulation of IL-18 mRNA and protein in the AD brain and CSF, and increased production of IL-18 by AD monocytes [34–36].

IL-33 is a member of IL-1 family that, in its precursor form, contains a nuclear localisation sequence that enables it to function as a transcription-regulating factor. IL-33 is released from producing cells as full-length protein upon cell death and is cleaved to shorter forms with classical cytokine activity [37]. As a cytokine, IL-33 binds to its receptor IL-1R4 (former name T1/ST2) and forms a signalling complex with IL-1R3, the promiscuous accessory chain of the IL-1 family. IL-33 initiates type 2-dependent inflammation and tissue repair, while in pathological situations it takes part in allergic and lung/mucosal inflammation [38–40]. The soluble receptor sIL-1R4 acts as an inhibitor of IL-33. In AD, IL-33 expression is decreased in the brain, and genetic polymorphisms of the *IL33* gene lead to a protective haplotype [41]. In an experimental model, IL-33 was found to ameliorate the AD-like pathology and cognitive decline [42].

The aim of the present study is to identify inflammation-related markers characteristic of the three disease conditions, which could be useful for prognostic and diagnostic purposes. This will be done through a comprehensive analysis of the levels of IL-1 family cytokines and receptors in the serum of SMC, MCI, AD, and cognitively healthy subjects (NHS). The results obtained could also provide meaningful information toward potential disease mechanisms, as a basis for the identification of future therapeutic targets.

## Methods

### Patients and controls

Two hundred and sixty subjects were consecutively recruited from the Centre for Research and Training in Medicine for Ageing at the University of Molise (Italy). Subjects were divided into four groups: 60 with probable AD (25 males, 35 females; mean age ± SD: 78.18 ± 8.44 years), 45 with amnestic MCI (19 males, 26 females; 66.98 ± 8.14 years), 61 with SMC (20 males, 41 females; 65.84 ± 6.43 years), and 94 NHS (41 males, 53 females; 68.65 ± 6.95 years). Table1 reports the clinical and demographic characteristics of the four groups of participants.

**Table 1:** Demographic and clinical features of the study subjects

	NHS	SMC	MCI	AD	*F* (3, 256)/*χ*^2#^	*p*
Demographic features						
Females/males [ratio n. subjects]	53/41	41/20	26/19	35/25	1.975	0.578
Age at study [mean years ± SD (range)]	68.64±6.95 (53–90)	65.84±6.43 (51–79)	66.98±8.14 (52–91)	78.13±8.35 (60–92)	34.14	< 0.001
Schooling [mean years ± SD]	11.11±4.06	11.80±4.11	10.31±4.51	7.08±5.00	4.16	< 0.001
BMI [mean ± SD]	28.21±4.29	28.09±4.67	29.21±4.36	25.73±4.34	6.278	< 0.001
Co-morbidities						
Smoking^+^ [n. subjects (%)]	15 (15.9%)	6 (9.8%)	6 (13.3%)	12 (20%)	2.618	0.454
Hypertension [n. subjects (%)]	44 (46.8%)	36 (59.0%)	24 (53.3%)	31 (51.6%)	2.252	0.522
Diabetes [n. subjects (%)]	13 (13.8%)	7 (11.5%)	6 (13.3%)	17 (28.3%)	8.015	0.046
Dyslipidemia [n. subjects (%)]	26 (27.6%)	9 (14.8%)	15 (33.3%)	23 (38.3%)	9.111	0.028
TIA/stroke [n. subjects (%)]	3 (3.2%)	6 (9.8%)	6 (13.3%)	8 (13.3%)	6.423	0.093
Myocardial infarction [n. subjects (%)]	6 (6.4%)	2 (3.3%)	4 (8.9%)	6 (10%)	2.451	0.484
Clinical manifestations						
MMSE < 24 [positive/negative]	0/94	0/61	2/43*	60/0	n.t.	< 0.001
MMSE [mean score ± SD]	29.12±1.30	29.44±1.3	28.54±1.82	12.15±7.70	289.33	< 0.001
Rey test failure [positive/negative]	0/94	0/61	32/13	60/0	n.t.	< 0.001
Pharmacological treatments						
Anti-hypertensive [n. subjects (%)]	49 (52.1%)	37 (60.6%)	22 (48.8%)	39 (65.0%)	3.985	0.263
Lipid-lowering [n. subjects (%)]	25 (26.6%)	15 (24.5%)	18 (40.0%)	20 (33.3%)	3.829	0.281
Hypoglycaemic [n. subjects (%)]	13 (13.8%)	6 (9.8%)	6 (13.3%)	18 (30.0%)	10.697	0.013
Antacids [n. subjects (%)]	22 (23.4%)	14 (22.9%)	13 (28.8%)	18 (30.0%)	1.319	0.725
Anti-platelets [n. subjects (%)]	23 (24.4%)	13 (21.3%)	11 (24.4%)	27 (45.0%)	10.685	0.014
Immunomodulatory [n. subjects (%)]	2 (2.1%)	2 (3.3%)	1 (2.2%)	1 (1.7%)	0.379	0.944
Anti-inflammatory [n. subjects (%)]	3 (3.2%)	2 (3.3%)	2 (4.4%)	0	2.357	0.502
Anti-gout [n. subjects (%)]	3 (3.2%)	2 (3.3%)	1 (2.2%)	1 (1.7%)	0.448	0.930
Supplements [n. subjects (%)]	19 (20.2%)	15 (24.5%)	4 (8.9%)	15 (25.0%)	5.118	0.163

^**#**^As described in the “Methods” section, *F* pertains to evaluation of age, schooling, BMI, and MMSE, while *χ*^2^ applies to all other parameters

^+^Current smokers

*Score 24 for both, i.e. borderline

*AD* Alzheimer’s disease, *BMI* body mass index, *MCI* mild cognitive impairment, *MMSE* Mini Mental State Examination, *NHS* normal healthy subjects, *SMC* subjective memory complaints, *TIA* transient ischemic attack, *n.t.* not tested

Patients with probable AD were diagnosed according to the National Institute on Ageing/Alzheimer’s Association (NIA-AA) criteria [43] and each fulfilled the criteria for the “probable AD with documented decline” category. They scored < 24 on the Mini Mental State Examination (MMSE) and > 0.5 on the Clinical Dementia Rating (CDR). Subjects with amnestic MCI met the NIA-AA diagnostic criteria for MCI due to AD [13], had an MMSE ≥ 24, had a CDR = 0.5, and showed memory impairment as assessed by age-sex-education-adjusted scores on at least one of the following two tests: Rey’s word list immediate and delayed recall, and Prose memory, immediate and delayed [44]. Participants with SMC stated that their memory function had deteriorated compared to earlier stages in life, reported that the time of onset was in adulthood, had a score of 24 or more on the Memory Complaint Questionnaire (MAC-Q) [45], and showed normal objective memory performance on Rey’s and Prose memory tests. To summarise, MCI subjects showed both subjective and objective memory impairment, SMC participants presented only memory complaints with a normal score on the memory tests, and NHS did not show either subjective or objective memory impairment. To rule out other potential causes of cognitive impairment, all participants underwent blood tests (including full blood count, erythrocyte sedimentation rate, urea and electrolytes, thyroid function, vitamin B12, and folate). Furthermore, all patients with AD and MCI, and 40 out of 61 subjects with SMC, underwent brain imaging. Depression was ruled out using the Geriatric Depression Scale-Short Form (GDS-SF) [46] and by excluding subjects with a GDS-SF score ≥ 6. The patients on treatment with cerebro-active drugs underwent a washout period of at least 14 days before assessment. Blood was collected by venipuncture, and serum samples were stored at − 80 °C until tested.

This study was conducted in accordance with ethical principles stated in the Declaration of Helsinki, as well as with approved national and international guidelines for human research. The Ethics Committee of University of Molise reviewed and approved this study, and a written informed consent was obtained from participants or caregivers.

### Evaluation of IL-1 family cytokines and receptors

Ten factors of the IL-1 family were examined, the agonist ligands IL-1α, IL-1β, IL-18, and IL-33, the antagonist ligand IL-1Ra, the soluble inhibitor IL-18BP, and the soluble receptors sIL-1R1, sIL-1R2, sIL-1R3, and sIL-1R4. A multiplex array was custom-made (Quansys Biosciences, Logan, UT, USA) and allowed us to use 50 μl of serum for simultaneously measuring all ten factors. The array was developed and optimised in order to reach excellent specificity, linearity, precision, and insensitivity to drift, interference, and edge effect. In addition, the array was validated for reproducibility of detection of each factor against single ELISA assays (R&D Systems, Minneapolis, MN). Reliable detection limits (linear part of the curve) were 1.8–500 pg/ml for IL-1α, 6–1500 for IL-1β, 4–8600 for IL-1Ra, 3.7–263 for IL-18, 0.9–641 for IL-33, 13–3070 for sIL-1R1, 2200–17,000 for sIL-1R2, 130–32,000 for sIL-1R3, 72–19,500 for sIL-1R4, and 580–146,000 for IL-18BP.

### Calculation of free IL-18

The circulating levels of free active IL-18, not bound and inhibited by its soluble inhibitor IL-18BP, were calculated as originally described by Novick et al. [47] and applied by Migliorini et al. [30]. Briefly, total IL-18 and IL-18BP were measured, having confirmed that measurements of each of the two factors are not interfered with by the presence of increasing amounts of the other factor. The calculation of free IL-18 was based on the molecular weight of the two interactors, the 1:1 type of interaction, and the *K*_d_ of 0.4 nM [48]. The law of mass action (*K*_d_ = [IL-18] × [IL-18BP]/[IL-18 × IL-18BP]) was applied using the following formula:

$$ x\kern0.5em =\kern0.5em \left(-\kern0.5em b\kern0.5em +\kern0.5em \surd {b}^2\kern0.5em -\kern0.5em 4c\right)/2 $$

in which *x* is the concentration of free IL-18, *b* is [IL-18] − [IL-18BP] + *K*_d_, and *c* is − *K*_d_ × [IL-18].

### Statistical analysis

Data are expressed as mean + SD. A *p* < 0.05 was considered statistically significant.

Data were analysed using the SPSS (v. 17.0) statistical software package (SPSS Inc., Chicago, IL, USA). Variables were examined for outliers and extreme values by using box and normal quantile-quantile plots, and with Shapiro-Wilk’s and Kolmogorov-Smirnov’s tests. When normal distribution was not achievable, mathematical transformation was applied (square, square root, logarithmic, reciprocal of square root, or reciprocal transformations were considered). However, normal distribution could not be achieved for IL-1α, IL-1β, IL-1Ra, and IL-33 values. Differences between groups in age, educational level, MMSE, and BMI were evaluated by one-way analysis of variance (ANOVA), while differences in sex, co-morbidities, and drug intake were evaluated with the chi-square test. Multiple regression analysis with dummy variables was applied to test for differences between NHS, SMC, MCI, and AD in the levels of IL-1 family cytokines and receptors and to evaluate the influence of several confounding variables (age, sex, educational level, BMI, MMSE, comorbidities, and drug therapy). Results of multiple linear regression, with *β* coefficients, 95% confidence intervals, and *p* values, for models with and without adjustment for confounding factors are reported in the text and in the supplementary materials (Additional files 1 and 2) so that the influence of confounding factors could be easily assessed. Correlation analysis was performed in each group and in the pooled group of patients (SMC, MCI, and AD) by Pearson’s correlation coefficient (*r*) for parametric variables and Spearman rank correlation coefficient (rs) for non-parametric variables, using Bonferroni’s correction for multiple comparisons. Stepwise multiple regression analysis was performed by considering the levels of IL-1 family cytokines and receptors as dependent variables and age, sex, educational level, BMI, MMSE, comorbidities, and drug therapy as independent variables. Results are reported as beta coefficients and 95% confidence intervals.

## Results

The levels of IL-1 family cytokines and receptors were measured in serum of AD patients and compared with those of MCI, SMC, and NHS. Because of significant differences between groups in age, schooling, body mass index, diabetes, dyslipidemias, and associated treatments with hypoglycaemic and anti-platelet drugs (Table 1), the statistical analysis took these differences into account by using multiple regression analysis with dummy variables (see Statistical analysis in the “Methods” section). Results of regression models without and with control for confounding variables are reported in the supplementary materials (Additional files 1 and 2).

**Fig. 1:**
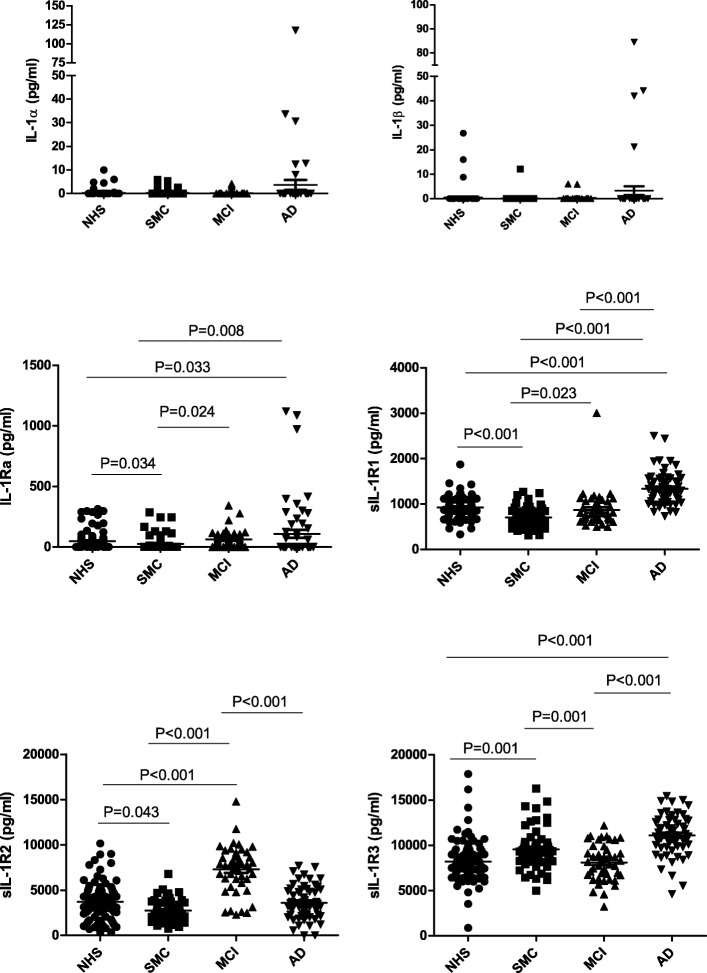
IL-1 ligands and soluble receptors in serum of patients with Alzheimer’s disease. The circulating levels of ligands and soluble receptors of the factors of the IL-1 sub-system were assessed in serum of normal healthy subjects (NHS) in comparison with those detected in serum of patients with subjective memory complaints (SMC), mild cognitive impairment (MCI), and patients with Alzheimer’s disease (AD). The factors tested are IL-1α (upper left), IL-1β (upper right), IL-1Ra (middle left), sIL-1R1 (middle right, sIL-1R2 (lower left), and sIL-1R3 (lower right). Statistically significant differences are indicated. All other comparisons are statistically not significant

First, we examined the IL-1 sub-system, which includes the agonist ligands IL-1α and IL-1β, the antagonist ligand IL-1Ra, and the soluble receptors sIL-1R1, sIL-1R2, and sIL-1R3 (Fig. 1). As expected, the levels of the inflammatory cytokines IL-1α and IL-1β were very low or undetectable in NHS (0.29 ± 1.37 pg/ml and 0.54 ± 3.31 pg/ml, respectively). Please note that these levels are below the reliable detection range of the multiplex array (see “Methods”). In patient groups, these levels remained low in SMC (0.30 ± 1.12 and 0.19 ± 1.55, respectively) and MCI (0.16 ± 0.76 and 0.26 ± 1.24, respectively) and increased in the AD group (3.6 ± 16.2 pg/ml and 3.2 ± 13.5 pg/ml, respectively), reaching statistical significance in the comparison AD vs. NHS (*p* = 0.018) for IL-1α, and AD vs. NHS (*p* = 0.014), AD vs. SMC (*p* = 0.046), and AD vs. MCI (*p* = 0.012) for IL-1β (Fig. 1, upper panels). The circulating levels of the antagonist IL-1Ra in AD (107.4 ± 246.9 pg/ml) were significantly higher than in NHS (49.2 ± 80.8 pg/ml, *p* = 0.049), but not vs. SMC (26.1 ± 65.7, *p* = 0.19) or MCI (64.1 ± 72.5, *p* = 0.14) (Fig. 1, centre left). When examining the presence of sIL-1R1 (the soluble form of the ligand-binding signalling receptor), it is interesting to see that its levels were significantly lower in SMC vs. NHS (706.8 ± 211.0 pg/ml vs. 925.7 ± 231.5 pg/ml, *p* < 0.001), but significantly higher in AD (1333.6 ± 365.6 pg/ml) as compared to all other groups, NHS (*p* = 0.009), SMC (706.8 ± 211.0 pg/ml, *p* < 0.001), and MCI (869.0 ± 388.2 pg/ml, *p* = 0.03) (Fig. 1, centre right). In the case of sIL-1R2, the classical soluble decoy receptor, its levels were significantly higher in MCI (7286.5 ± 2599.5 pg/ml) compared to other groups (3715.6 ± 2004.2 pg/ml in NHS, 2754.8 ± 1113.1 pg/ml in SMC, and 3603.9 ± 1820.9 pg/ml in AD; *p* < 0.001) (Fig. 1, lower left). For the soluble form of the accessory chain sIL-1R3, its levels were significantly higher in AD (11,038.0 ± 2278.1 pg/ml) compared to NHS (8275.4 ± 2158.7 pg/ml, *p* = 0.029) and MCI (8096.7 ± 2012.0 pg/ml, *p* = 0.016), and in SMC (9548.6 ± 2169.0 pg/ml) compared to NHS (*p* < 0.001).

**Fig. 2:**
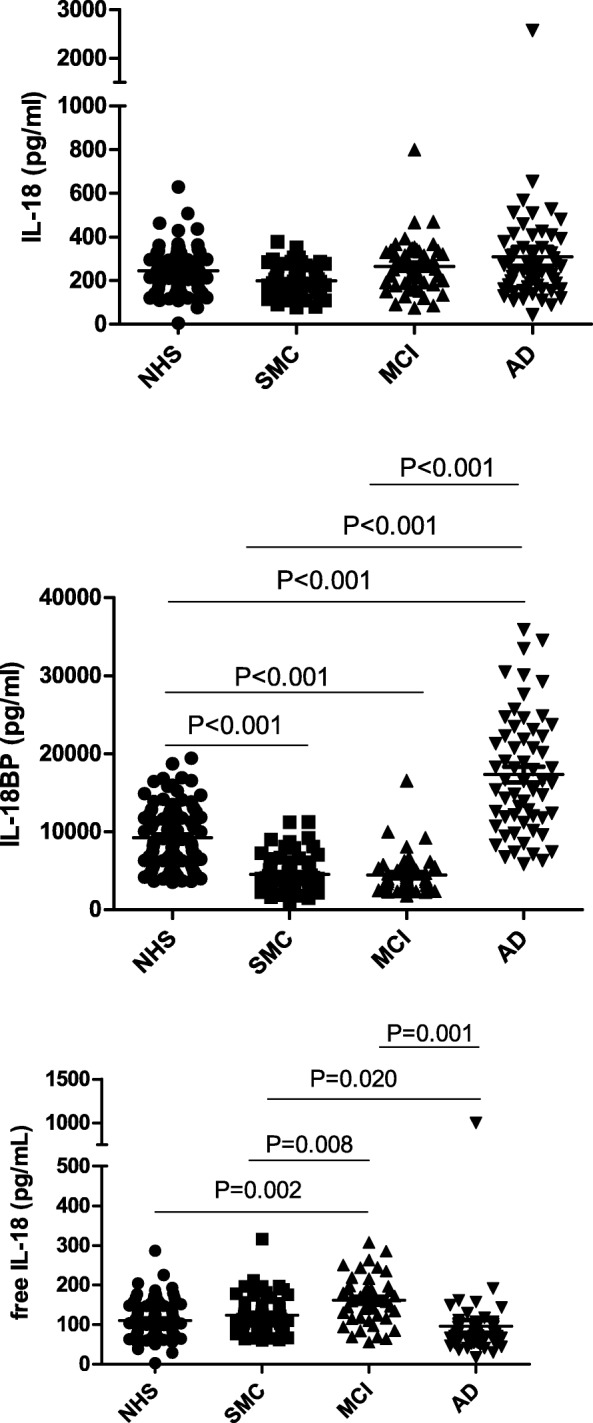
IL-18 and IL-18BP in serum of patients with Alzheimer’s disease. The circulating levels of ligands and soluble receptors of the factors of the IL-18 sub-system were assessed in serum of normal healthy subjects (NHS) in comparison with those detected in serum of patients with subjective memory complaints (SMC), mild cognitive impairment (MCI), and patients with Alzheimer’s disease (AD). The factors tested are IL-18 (upper panel) and IL-18BP (middle panel). In the lower panel are reported the values of free IL-18, calculated from the quantitative values of IL-18 and IL-18BP. Statistically significant differences are indicated. All other comparisons are statistically not significant

We also examined the IL-18 sub-system, by evaluating the levels of the cytokine IL-18 and of its inhibiting protein IL-18BP and by calculating the levels of inhibitor-free IL-18. As shown in Fig. 2 (upper panel), the levels of total IL-18 were significantly lower in SMC (199.0 ± 67.8 pg/ml) compared to NHS (245.6 ± 96.0 pg/ml, *p* = 0.01) and MCI (264.8 ± 128.0 pg/ml, *p* = 0.019), but not AD (309.8 ± 324.3 pg/ml). Regarding IL-18BP (Fig. 2, centre panel), it is notable that the levels of the inhibitor were significantly lower in both SMC (4512.7 ± 2387.7 pg/ml) and MCI (4450.0 ± 2592.0 pg/ml) compared to NHS (9172.2 ± 3984.8 pg/ml, *p* < 0.001), but significantly higher in AD (17,265.0 ± 7528.4 pg/ml) vs. NHS (*p* = 0.001). By calculating free IL-18 (Fig. 2, lower panel), subjects with MCI (161.6 ± 60.2 pg/ml) showed a significant increase in the levels of the free cytokine compared to NHS (111.0 ± 45.1 pg/ml, *p* < 0.001), SMC (124 ± 45.8 pg/ml, *p* = 0.012), and AD (95.4 ± 123.4 pg/ml, *p* = 0.049).

**Fig. 3:**
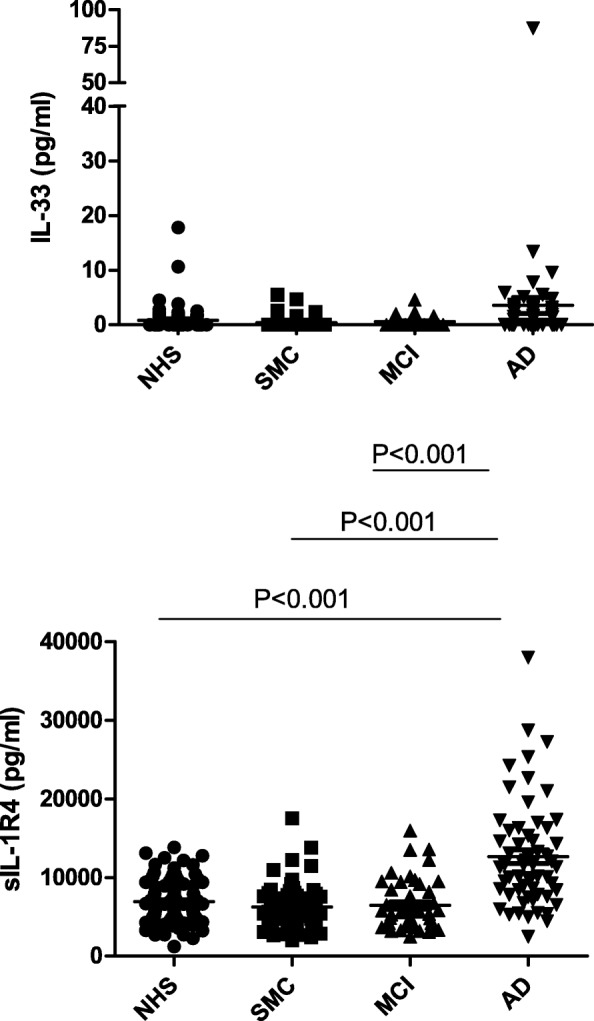
IL-33 and soluble receptor in serum of patients with Alzheimer’s disease. The circulating levels of ligands and soluble receptors of the factors of the IL-33 sub-system were assessed in serum of normal healthy subjects (NHS) in comparison with those detected in serum of patients with subjective memory complaints (SMC), mild cognitive impairment (MCI), and patients with Alzheimer’s disease (AD). The factors tested are IL-33 (upper panel) and sIL-1R4 (lower panel). Please note that the third component of the sub-system, the soluble accessory receptor chain sIL-1R3, is already reported in Fig. 1, as it is common to both the IL-1 and the IL-33 sub-systems. Statistically significant differences are indicated. All other comparisons are statistically not significant

The third sub-system examined is that of IL-33 and its soluble receptor sIL-1R4. It should be emphasised that sIL-1R3 (Fig. 1, lower left) is also part of the IL-33 sub-system, as IL-1R3 is the accessory chain for both IL-1R1 and IL-1R4. The circulating levels of IL-33 are in general very low, with only a limited increase in AD (3.5 ± 11.3 pg/ml) that, however, reaches statistical significance in comparison with NHS (0.85 ± 2.2 pg/ml, *p* = 0.006), SMC (0.40 ± 1.05, *p* = 0.043), and MCI (0.60 ± 0.91, *p* = 0.014) (Fig. 3, upper panel). The average IL-33 levels in NHS, SMC, and MCI are all below the reliable detection level of the multiplex array (see “Methods”). In the case of sIL-1R4, SMC and MCI patients were not different from NHS, whereas sIL-1R4 levels in AD patients (12,546.7 ± 6791.1 pg/ml) showed a significant increase vs. all other groups, NHS (6922.7 ± 2687.0 pg/ml, *p* = 0.028), SMC (6274.4 ± 2915.1 pg/ml, *p* = 0.023), and MCI (6450.8 ± 3231.4 pg/ml, *p* = 0.034) (Fig. 3, lower panel).

Correlation analysis showed a significant positive correlation of sIL-1R1 (*r* = 0.46, *p* < 0.001), sIL-1R4 (*r* = 0.42, *p* < 0.001), and IL-18BP (*r* = 0.64, *p* < 0.001) with age in the NHS group, and between IL-18BP (*r* = 0.40, *p* = 0.001) and age in the SMC group. There was no correlation between IL-1 family mediators and drug treatment, including anti-hypertensive or lipid-lowering drugs, or co-morbidities. In the pooled group of patients (SMC, MCI, and AD), correlation analysis showed a significant negative correlation between the MMSE scores and IL-18 (*r* = − 0.223, *p* = 0.004), sIL-1R1 (*r* = − 0.583, *p* < 0.001), sIL-1R4 (*r* = − 0.489, *p* < 0.001), IL-18BP (*r* = − 0.663, *p* < 0.001), sIL-1R3 (*r* = − 0.359, *p* < 0.001), and IL-33 (rs = − 0.333, *p* < 0.001). Multiple regression analysis showed that correlation with MMSE was significant only for sIL-1R1 (− 17.93; − 24.09 to − 11.78; *p* < 0.001), sIL-1R4 (− 224.7; − 316.37 to − 133.04; *p* < 0.001), IL-18BP (− 401.67; − 505.51 to − 297.85; *p* < 0.001), and sIL-1R3 (− 93.14; − 130.45 to − 55.82; *p* < 0.001), while correlation with age was significant for sIL-1R1 (15.76; 9.63 to 21.88; *p* < 0.001), sIL-1R4 (122.82; 31.64 to 214.06; *p* = 0.009), and IL-18BP (288.2; 184.92 to 391.49; *p* < 0.001).

## Discussion

The results of a comprehensive analysis of the circulating levels of IL-1 family cytokines and receptors reveal that AD is characterised by a significant increase in the levels of many of these proteins. This supports the hypothesis that an important inflammatory status is associated with AD, along with the consequent activation of a number of control mechanisms. The high circulating levels of IL-1 factors, in particular the inhibitory molecules, imply a long-range control system that effectively dampens tissue-localised inflammation. We have summarised in Table 2 the results obtained in this study of AD, MCI, and SMC subjects, and compared them with data pertaining to other diseases, in which the cytokines and factors of the IL-1 family have been measured with the same or comparable assays.

**Table 2:** Variations of IL-1 family cytokines and receptors in AD patients and other diseases *vs.* healthy controls

IL-1 family molecule	Disease					
	AD	MCI	SMC	ALS	SLE	IgG4-RD
*IL-1 subsystem*						
IL-1α	↑	=	=	n.t.	=	=
IL-1β	↑	=	=	=	=	=
IL-1Ra	↑	=	=	=	=	=
sIL-1R1	↑	=	↓	n.t.	=	↑
sIL-1R2	=	↑	↓	=	=	↑
sIL-1R3	↑	=	↑	n.t.	=	↓
*IL-18 subsystem*						
IL-18	=	=	↓	↑	↑	=
IL-18BP	↑	↓	↓	↑	↑	=
Free IL-18	=	↑	=	↑	↑	=
*IL-33 subsystem*						
IL-33	↑	=	=	=	↓	=
sIL-1R4	↑	=	=	=	↑	↑
sIL-1R3	↑	=	↑	n.t.	=	↓

Results for ALS, SLE, and IgG4-related disease (RD) are from references [28, 29, 31–33]

*n.t.* not tested

A limitation of our study is the differences in age, comorbidity, and treatments between healthy subjects and AD patients. Consequently, some of the changes in the levels of inflammatory mediators might depend on age, rather than AD, co-presence of another disease, such as diabetes, or pharmacological treatment, such as antiplatelet or hypoglycaemic drugs. Therefore, the comparison of variables between groups was made by multiple regression analysis with dummy variables, in order to adjust for age, presence of comorbidities, and drug treatment (Additional files 1 and 2). Nevertheless, additional analysis with a fully matched population in a longitudinal study is needed to fully validate the role of these inflammatory mediators in the pathogenesis of AD.

IL-1α and IL-1β, which are potent bioactive cytokines, are generally undetectable in human circulation, both in normal and pathological conditions, as their activity is usually confined to tissue locations where an inflammatory reaction is taking place. The same was observed in this study, in which IL-1α and IL-1β levels were low/undetectable in healthy controls, and also in patients with SMC and MCI, with the exception of AD patients who showed a small but statistically significant increase in the levels of both cytokines. Conversely, high levels of the soluble forms of the two IL-1 receptors, sIL-1R1 and sIL-1R2, and of their accessory receptor sIL-1R3 are observed in normal conditions. It is likely that these exist in circulation as a reservoir for the regulation of IL-1 activity at the tissue level. Two of these receptors are increased in AD (sIL-1R1 and sIL-1R3), one in SMC (sIL-1R3), and one in MCI (sIL-1R2). This finding suggests that a more forceful downregulation of IL-1 signalling is required in patients’ tissues. That upregulation of soluble receptors differs among groups is suggestive of differences in the mechanisms of IL-1-dependent inflammation. In the case of AD, there is an increase in sIL-1R1 but not sIL-1R2. The increase in sIL-1R1 suggests that the underlying inflammatory reaction has selectively activated the IL-1R1-cleaving protease. We should be aware that this is not the most efficient mechanism for inhibiting IL-1; sIL-1R2 is a much more potent IL-1 inhibitor. In fact, sIL-1R1 is also bound with high affinity by IL-1Ra (thereby decreasing its capacity to capture IL-1), while sIL-1R2 nearly exclusively binds to IL-1. The fact that the circulating levels of IL-1Ra are increased in AD patients suggests that a large part of the IL-1-inhibiting capacity of sIL-1R1 is in fact blocked, leaving AD patients with less capacity to control IL-1-induced inflammation.

The inhibitory role of sIL-1R3 is only partially known [28]. This soluble receptor is generated by alternative splicing (not by proteolytic cleavage of the membrane receptor) and is able to increase the binding affinity of sIL-1R2 for capture of IL-1. A similar role of sIL-1R3 has been hypothesised, but not proven, in which sIL-1R3 is expected to improve IL-1 capture by sIL-1R1 and also IL-33 capture by sIL-1R4 [19]. Therefore, the increase of circulating sIL-1R3 in AD and SMC subjects (while sIL-1R2 levels remain constant in AD and decreases in SMC) would suggest an increasing efficiency of IL-1 capture and inhibition by the available sIL-1R2. Notably, in MCI there is no increase in sIL-1R3 but a significant increase in sIL-1R2, thereby achieving the same result (more efficient IL-1 capture/inhibition), albeit with a different strategy. An increase in the levels of the other IL-1 inhibitor, the receptor antagonist IL-1Ra, is evident only in AD patients and underlines the attempt of the organism to inhibit IL-1-dependent inflammation by multiple strategies. However, as mentioned above, it is likely that the two inhibitors sIL-1R1 and IL-1Ra bind each other, and thus neutralise their inhibitory effect. An increase in circulating IL-1Ra is not a finding common to many diseases (see Table 2), and it has been mainly observed in severe inflammatory conditions such as chronic arthritis and sepsis.

In inflammation-based diseases, an increase in circulating IL-18 is often observed [27]. However, this increase is regulated by a concomitant increase of the inhibitor IL-18BP, with the purpose of returning free active IL-18 to normal background levels [28–33]. In AD patients, we observed a slight increase in circulating IL-18 levels, which however did not reach statistical significance. No difference from controls was observed in MCI subjects. This is in agreement with previous data that show no significant increase in the circulating levels of IL-18 in either AD or MCI patients [5, 35, 49–51]. However, other publications report an increase of IL-18 in AD [52, 53], with some authors suggesting a direct correlation between IL-18 levels and disease severity [54], while others showed an inverse correlation [55]. In our AD patient cohort, IL-18 levels and MMSE score showed a weak, but significant correlation, which however disappeared after multiple regression analysis. In the absence of an increase in IL-18 levels, the increase in IL-18BP levels in AD patients is surprising. It should be noted, however, that the levels of free IL-18 (i.e. the fraction of IL-18 that is not blocked by IL-18BP and is therefore free to exert its biological activity) is significantly increased in MCI compared to other groups, due to a significant decrease in the IL-18BP levels. A decrease in IL-18BP is evident in both SMC and MCI, although the consequent increase in the levels of free IL-18 reaches statistical significance only for MCI. This would suggest a role for IL-18 in the pathogenesis of AD, as the increase of free IL-18 in MCI may imply early IL-18-dependent inflammation-driven alterations in the blood-brain barrier and neuronal damage [56], preceding the development of AD. In AD, increased expression and production of IL-18 in the brain may be the cause of a general and systemic increase of its inhibitor [34, 35], in a feedback mechanism resulting in a reduction of free IL-18 levels. Thus, the levels of free IL-18, which are significantly elevated in both SMC and MCI conditions, are reduced in AD patients and comparable to healthy controls.

The alarmin IL-33 is almost undetectable in the circulation of healthy individuals, as well as in SMC and MCI patients, yet it increases significantly in AD patients. IL-33 is a “dual” cytokine involved both in non-classical type 2 inflammation and in tissue repair and homeostatic metabolic control [38]. The finding that IL-33 is cardioprotective has led to classifying the IL-33 inhibitor sIL-1R4 as a biomarker of cardiac stress and a risk factor for heart failure [57]. Since there are data suggesting that IL-33 can also be neuroprotective and associated with decreased AD risk [42, 43], we may infer that sIL-1R4 could also be a biomarker for increased AD risk. However, elevated levels of sIL-1R4 have been found in several diseases [33, 34], and as reaction to mechanical stress (PI and DB, unpublished observations), without implying risk of cardiac failure or AD. This suggests that sIL-1R4 is a general marker of inflammation [58]. Circulating levels of sIL-1R4 are significantly elevated in AD patients compared to healthy controls, SMC and MCI subjects, further supporting the hypothesis of an ongoing inflammation condition.

From the summary presented in Table 2, it is notable that many of the factors that are increased in AD are inflammation-inhibiting molecules: the IL-1 inhibitors IL-1Ra, sIL-1R1, and sIL-1R3, the IL-18 inhibitor IL-18BP, and the IL-33 inhibitors sIL-1R4 and sIL-1R3. The increase of IL-33 levels, although limited, can be considered an endogenous attempt at neuroprotection. Likely, the high levels of anti-inflammatory factors in the circulation are a mechanism for controlling inflammation at tissue sites. The fact that several anti-inflammatory factors are increased underlines the redundancy of the control system and the intensive effort of the organism to downregulate pathological inflammation. The increased levels of all these factors observed in AD patients therefore imply increased and uncontrollable tissue-localised inflammation.

In SMC subjects, changes compared to controls are limited to an increase in sIL-1R3 and a decrease in sIL-1R1, sIL-1R2, IL-18, and IL-18BP levels. All these factors are anti-inflammatory, with the exception of IL-18. The increase in the sIL-1R3 levels would likely translate in an increased efficiency of sIL-1R2 in capturing and inhibiting IL-1 at the tissue level. The decrease in IL-18BP levels is in line with the elevated levels of free IL-18 in SMC that however did not reach statistical significance. The decrease in sIL-1R1 may imply a reduced capacity of inhibiting IL-1 at the tissue level; however, this seems to be replaced by a more efficient sIL-1R2/sIL-1R3 mechanism.

In MCI patients, changes compared to healthy individuals are limited but peculiar. IL-18BP is decreased, as observed also in SMC, but in this case, the decrease leads to a significant increase in free active IL-18, meaning that an increase in active IL-18 may be occurring. IL-18 has numerous functions, in particular in the stimulation of both innate and adaptive immune responses [26–28]. Higher IL-18 activity would therefore imply an endogenous demand for enhanced immune defences. It is very interesting that in MCI there is a significant increase in sIL-1R2. This is the classical and most effective inhibitor of IL-1, with excellent anti-inflammatory activity. Increased sIL-1R2 levels are symptomatic of IL-1-dependent inflammation taking place in tissues, to which the organism responds by increasing the levels of the most effective inhibitor.

In AD, the levels of sIL-1R2 are similar to those of healthy controls, while other IL-1 inhibitors are increased, a finding that implies a significant disease-driven alteration of immune status and, consequently, a change in the mechanisms used by the organism in the attempt to control inflammation. As mentioned above, the diverse inhibitory mechanisms activated in AD may imply an increased need of controlling inflammation. It is again worth mentioning that some of these inhibitory molecules neutralise each other (as in the case of the IL-1 inhibitor IL-1Ra binding to the IL-1 inhibitor sIL-1R1), practically hampering the capacity of the organism to control inflammation and the ensuing pathology.

To summarise the progression from SMC to AD dementia, SMC is characterised by an increase in sIL-1R3 and a decrease in sIL-1R1, sIL-1R2, IL-18, and IL-18BP. MCI does not demonstrate an increase in sIL-1R3 or a decrease in sIL-1R1 but does show decreased IL-18BP, and a peculiar increase in sIL-1R2. AD displays a characteristic increase in IL-1Ra (not observed in other conditions), in sIL-1R1 (observed in IgG4-related diseases), in sIL-1R3 (as in SMC), in IL-18BP (observed in other diseases, but not in SMC or MCI that show a decrease), and in sIL-1R4. Thus, AD patients show a characteristic profile of increased IL-1 family-related factors, which is different from that of other diseases and, most importantly, very different from MCI. The differences between MCI and AD are the normalisation of sIL-1R2, the reversal of IL-18BP, and the increase in IL-1α, IL-1β, IL-1Ra, sIL-1R1, sIL-1R3, IL-33, and sIL-1R4.

## Conclusions

Our analysis of circulating levels of IL-1 family cytokines and receptors in AD patients demonstrates significant increases of almost all the anti-inflammatory effectors, in parallel to a small increase in the inflammatory cytokines IL-1α and IL-1β. This finding implies the presence of active and persistent inflammation at the tissue level, to which the organism responds by activating multiple control mechanisms, albeit inefficiently (i.e. by failing to increase sIL-1R2, alongside the mutual inactivation of IL-1Ra and sIL-1R1). AD patients show a characteristic profile of increased IL-1α, IL-1β, IL-1Ra, sIL-1R1, sIL-1R3, IL-33, sIL-1R4, and IL-18BP. It is notable that sIL-1R1, sIL-1R3, sIL-1R4, and IL-18BP show a strong negative correlation with severity of cognitive impairment as assessed by MMSE. Interestingly, MCI subjects (46% of which will progress to AD dementia in 3 years) have normal levels of all these factors except for IL-18BP (lower than in healthy controls), while demonstrating increased free IL-18 and sIL-1R2, which are normal in AD patients. The distinct profiles of MCI vs. AD suggest the possibility of using these factors as biomarkers for following the disease progression.

## Additional files

Additional file 1: Results of multiple linear regression models without control for confounding variables. Values are β coefficients (95% confidence intervals) and *p* values. AD, Alzheimer’s disease; MCI, mild cognitive impairment; NHS, normal healthy subjects; SMC, subjective memory complaint. (PDF 78 kb)

Additional file 2: Results of multiple linear regression models with control for confounding variables. Values are β coefficients (95% confidence intervals) and *p* values. AD, Alzheimer’s disease; MCI, mild cognitive impairment; NHS, normal healthy subjects; SMC, subjective memory complaint. (PDF 75 kb)

## Abbreviations

AD: Alzheimer’s disease
ALS: Amyotrophic lateral sclerosis
BMI: Body mass index
CSF: Cerebrospinal fluid
MCI: Mild cognitive impairment
MMSE: Mini Mental State Examination
NHS: Normal healthy subjects
SLE: Systemic lupus erythematosus
SMC: Subjective memory complaints
TIA: Transient ischemic attack
