# Supplementary material for: Circulating levels of IL-1 family cytokines and receptors in Alzheimer’s disease: new markers of disease progression?
Source: J Neuroinflammation. 2018 Dec 12;15:342. doi: 10.1186/s12974-018-1376-1 (PMC6292179; doi:10.1186/s12974-018-1376-1)
Supplement: Supplementary file 1 — Results of multiple linear regression models without control for confounding variables. Values are β coefficients (95% confidence intervals) and p values. AD, Alzheimer’s disease; MCI, mild cognitive impairment; NHS, normal healthy subjects; SMC, subjective memory complaint. (PDF 78 kb) [file 12974_2018_1376_MOESM1_ESM.pdf]

Additional file 1. Results of multiple linear regression models without control for confounding variables

	**NHS *vs.* SMC**	**NHS *vs.* MCI**	**NHS *vs.* AD**	**AD *vs.* SMC**	**AD *vs.* MCI**	**MCI *vs.* SMC**
IL-1α	0.1 (-0.40 to 0.42)<br>*p*=0.96	-0.13 (-0.56 to 0.3)<br>*p*=0.54	3.29 (-0.29 to 6.61)<br>*p*=0.052	3.28 (-0.84 to 7.41)<br>*p*=0.12	3.42 (-1.38 to 8.23)<br>*p*=0.16	-0.14 (-0.53 to 0.24)<br>*p*=0.46
IL-1β	-0.35 (-1.2 to 0.54)<br>*p*=0.44	-0.28 (-1.29 to 0.72)<br>*p*=0.57	2.6 (-0.21 to 5.51)<br>*p*=0.07	3.0 (-0.43 to 6.43)<br>*p*=0.86	2.9 (-1.06 to 6.93)<br>*p*=0.14	0.06 (-0.49 to 0.62)<br>*p*=0.81
IL-1Ra	-23.1 (-47.5 to 1.2)<br>*p*=0.06	14.8 (-13.1 to 42.9)<br>*p*=0.29	58.1 (3.8 to 112.4)<br>*p*=0.036	81.3 (16.5 to 146.1)<br>*p*=0.014	43.2 (-32.1 to 118.6)<br>*p*=0.26	38.0 (11.2 to 64.8)<br>*p*=0.006
IL-18	-46.6 (-74.5 to -18.6)<br>*p*=0.001	19.1 (-19.3 to 57.6)<br>*p*=0.32	64.2 (-6.1 to 134.6)<br>*p*=0.073	110.8 (26.8 to 194.8)<br>*p*=0.010	45.02 (-56.3 to 146.7)<br>*p*=0.38	65.8 (27.6 to 103.9)<br>*p*=0.001
IL-33	-0.45 (-1.05 to 0.15)<br>*p*=0.14	-0.25 (-0.93 to 0.43)<br>*p*=0.46	2.6 (0.29 to 5.0)<br>*p*=0.028	3.1 (0.23 to 5.9)<br>*p*=0.034	2.9 (-0.43 to 6.2)<br>*p*=0.088	0.19 (-0.19 to 0.58)<br>*p*=0.31
sIL-1R1	-218.9 (-291.5 to -146.2)<br>*p*<0.001	-56.6 (-161.06 to 47.7)<br>*p*=0.28	407.8 (312.9 to 502.86)<br>*p*<0.001	626.8 (519.5 to 734.04)<br>*p*<0.001	464.52 (317.6 to 611.3)<br>*p*<0.001	162.2 (45.74 to 278.8)<br>*p*=0.007
sIL-1R2	-961.1 (-1516.8 to -405)<br>*p*=0.001	3570.6 (2777.3 to 4363.8)<br>*p*<0.001	-112.0 (-743.8 to 519.6)<br>*p*<0.726	849.0 (306.7 to 1391.2)<br>*p*=0.002	-3682.6 (-4538.2 to -2827)<br>*p*<0.001	4531.7 (3795.0 to 5268.3)<br>*p*<0.001
sIL-1R3	1273.1 (570.6 to 1975.6)<br>*p*<0.001	-178.7 (-936.0 to 578.6)<br>*p*<0.64	2762.5 (2042.4 to 3482.7)<br>*p*<0.001	1489.4 (688.8 to 2290.0)<br>*p*<0.001	2941.2 (2093.1 to 3789.3)<br>*p*<0.001	-1451.8 (-2271.4 to -631.9)<br>*p*=0.001
sIL-1R4	-648.3 (-1550.9 to 254.2)<br>*p*=0.158	-471.9 (-1501.8 to 557.9)<br>*p*=0.336	5623.9 (4081.6 to 7166.3)<br>*p*<0.001	6272.3 (4396.3 to 8148.3)<br>*p*<0.001	6095.8 (3922.5 to 8269.1)<br>*p*<0.001	176.4 (-1013.2 to 1366.1)<br>*p*=0.76
IL-18BP	-4659 (-5579 to -3539.6)<br>*p*<0.001	-4722.2 (-6011 to -3342.9)<br>*p*<0.001	8092.7 (6254 to 9931)<br>*p*<0.001	12752.3 (10748.5 to 14756)<br>*p*<0.001	12815 (10490.1 to 15139)<br>*p*<0.001	-62.6 (-1027-6 to 902.2)<br>*p*=0.89
free IL-18	13.18 (-1.55 to 27.92)<br>*p*=0.79	50.51 (32.43 to 68.6)<br>*p*<0.001	-15,60 (-43.27 to 11.98)<br>*p*=0.26	-28.8 (-62.2 to 4.57)<br>*p*=0.09	-66.16 (-105.8 to -26.5)<br>*p*=0.001	37.33 (16.93 to 57.7)<br>*p*<0.001

Values are β coefficients (95% confidence intervals) and *p* values.

AD, Alzheimer's disease; MCI, mild cognitive impairment; NHS, normal healthy subjects; SMC, subjective memory complaint.
