# Supplementary material for: Circulating levels of IL-1 family cytokines and receptors in Alzheimer’s disease: new markers of disease progression?
Source: J Neuroinflammation. 2018 Dec 12;15:342. doi: 10.1186/s12974-018-1376-1 (PMC6292179; doi:10.1186/s12974-018-1376-1)
Supplement: Supplementary file 2 — Results of multiple linear regression models with control for confounding variables. Values are β coefficients (95% confidence intervals) and p values. AD, Alzheimer’s disease; MCI, mild cognitive impairment; NHS, normal healthy subjects; SMC, subjective memory complaint. (PDF 75 kb) [file 12974_2018_1376_MOESM2_ESM.pdf]

Additional file 2. Results of multiple linear regression models with control for confounding variables

	**NHS *vs.* SMC**	**NHS *vs.* MCI**	**NHS *vs.* AD**	**AD *vs.* SMC**	**AD *vs.* MCI**	**MCI *vs.* SMC**
IL-1α	0.19 (-0.25 to 0.63) *p*=0.39	-0.21 (-0.68 to 0.24) *p*=0.35	9.17 (1.62 to 16.7) *p*=0.018	7.9 (-1.81to 17.6) *p*=0.11	9.97 (-0.69 to 20.6) *p*=0.06	-0.13 (-0.61 to 0.34) *p*=0.58
IL-1β	-0.15 (-1.1 to 0.8) *p*=0.75	-0.4 (-1.58 to 0.59) *p*=0.37	7.9 (1.63 to 14.3) *p*=0.014	8.1 (0.16 to 16.1) *p*=0.046	11.1 (2,51 to 19.7) *p*=0.012	0.23 (-0.34 to 0.8) *p*=0.42
IL-1Ra	-25.9 (-52.5 to 0.6) *p*=0.056	-3.7 (-33.9 to 26.5) *p*=0.8	116.9 (0.34 to 237.3) *p*=0.049	98.3 (-51.8 to 248.5) *p*=0.19	125.3 (-42.2 to 293.3) *p*=0.14	40.9 (7.6 to 74.2) *p*=0.016
IL-18	-39.4 (-69.4 to -9.3) *p*=0.01	16.7 (-22.2 to 55.7) *p*=0.39	-48.6 (-206.8 to 109.5) *p*=0.54	-16.1 (-210.2 to 178.1) *p*=0.87	-40.6 (-259.9 to 178.7) *p*=0.71	52.8 (8.8 to 96.8) *p*=0.019
IL-33	-0.39 (-1.09 to 0.29) *p*=0.26	-0.60 (-1.38 to 0.17) *p*=0.12	7.5 (2.2 to 12.9) *p*=0.006	6.9 (0.21 to 13.6) *p*=0.043	9.1 (1.8 to 16.4) *p*=0.014	0.25 (-0.22 to 0.741) *p*=0.29
sIL-1R1	-193.4 (-266.5 to -120.4) *p*<0.001	-58.2 (-155.2 to 38.82) *p*=0.23	268.9 (68.41to -469.58) *p*=0.009	471.2 (242.1 to 700.3) *p*<0.001	327.2 (31.57 to 622.9) *p*=0.03	81.65 (-36.6 to 199.9) *p*=0.173
sIL-1R2	-812.8 (-1383.2 to -242.4) *p*=0.006	3402.4 (2567.4 to 4237.4) *p*<0.001	-1337.8 (-2733.4 to 57.8) *p*=0.06	212.5 (-1061.0 to 1486.1) *p*=0.74	-4148.5 (-6065.7 to -2231) *p*<0.001	4098.8 (3154.7 to 5042.9) *p*<0.001
sIL-1R3	1526.13 (759.1 to 2293.0) *p*<0.001	-255.9 (-1122.8 to 610.9) *p*=0.56	1765.6 (180.85 to 3350.5) *p*=0.029	607.3 (-1258.0 to 2472.6) *p*=0.52	2306.2 (435.3 to 4177.3) *p*=0.016	-947.8 (-1932.6 to 36.8) *p*=0.059
sIL-1R4	-53.0 (-946.5 to 840.4) *p*=0.9	-594.0 (-1627.4 to 439.4) *p*=0.25	3761.7 (422.1 to 7101.4) *p*=0.028	4780.1 (658.9 to 8901.3) *p*=0.023	5005.9 (379.3 to 9632.6) *p*=0.034	114.2 (-1276.0 to 1504.5) *p*=0.87
IL-18BP	-3848.2 (-4831.3 to -2865) *p*<0.001	-4742.6 (-5906 to -3759.4) *p*<0.001	6792.1 (2938.5 to 10645.5) *p*=0.001	12377.6 (7917.1 to 16838) *p*<0.001	13298.1 (8389.8 to 18206) *p*<0.001	109.79 (-828.7 to 1048.3) *p*=0.81
free IL-18	10.99 (-4.79 to 26.79) *p*=0.17	48.98 (29.74 to 68.2) *p*<0.001	-47.0 (-110.64 to 16.45) *p*=0.14	-69.5 (-148.0 to 9.03) *p*=0.082	-88.73 (-177.0 to -0.39) *p*=0.049	33.0 (7.49 to 58.56) *p*=0.012

Values are β coefficients (95% confidence intervals) and *p* values.

AD, Alzheimer's disease; MCI, mild cognitive impairment; NHS, normal healthy subjects; SMC, subjective memory complaint.
